# The clinical significance of anti-CCP antibodies in Korean JRA patients

**DOI:** 10.1186/1546-0096-12-S1-P198

**Published:** 2014-09-17

**Authors:** Kwang Nam Kim, Jung Woo Rhim

**Affiliations:** 1 Pediatrics, Hallym University Medical Center, An-Yang, Korea, Republic Of; 2 Pediatrics, The Catholic Medical Center, Dae Jeon, Korea, Republic Of

## Introduction

To determine the clinical significance of anti-CCP (anti-citrullinated cyclic peptide) in Korean patients with JRA. Rheumatoid Factor (RF) is a non-specific serologic test in juvenile rheumatoid arthritis (JRA) but the anti-perinuclear autoantibodies are found in adult rheumatoid arthritis (RA) recently. One of these autoantibodies can detect epitopes called citrulline. The authors want to know that these autoantibodies can be applied in Korean JRA.

## Objectives

The serum samples were obtained from 142 patients with arthralgia. The mean ages are 8.98 years old. The case group were 83 patients with JRA and the other control group were 59 patients with arthralgia who are not JRA.

## Methods

The ant-CCP antibodies were assessed by an enzyme linked immunosorbent assay (ELISA) : INOVA Quant Lit CCP3 IgG ELISA test.

## Results

The authors compared the anti-CCP values with each JRA subtypes. Each subtypes are systemic JRA (29 cases), pauci JRA (31 cases) and poly JRA (23 case). The p-values are 1.0, 0.272 and <0.01 respectively. We are also compared the anti-CCP values with RF-positive and RF-negative in poly JRA. The p-values are <0.01 and <0.01 both.

## Conclusion

The authors compared the JRA group with the control group. The p-value revealed <0.01 significantly. We also compared each subtype of JRA with control group. Only the poly JRA group is significantly differences with control group (p<0.01). We concluded that the test for anti-CCP in poly JRA patients are significantly benefit in the clinical field.

## Disclosure of interest

None declared.

